# Association of rs11644461 GRIN2A with clinical phenotype of schizophrenia

**DOI:** 10.1192/j.eurpsy.2023.763

**Published:** 2023-07-19

**Authors:** E. Poltavskaya, O. Fedorenko, E. Kornetova, I. Pozhidaev, D. Paderina

**Affiliations:** Tomsk National Research Medical Center, Mental Health Research Institute, Tomsk, Russian Federation

## Abstract

**Introduction:**

The glutamatergic system plays an important role in the neurobiology of schizophrenia. A lot of number of variants in the GRIN genes have been found in patients with various neuropsychiatric disorders (Myers et al. F1000Res 2019; 8(F1000 Faculty Rev) 1940). GluN2A, encoded by the GRIN2A gene, is the most abundant of the GluN2 NMDA receptor subunits in the mammalian CNS. Clinical symptoms of schizophrenia vary among individuals. The GRIN2A gene has previously been shown to be associated with early onset schizophrenia (Poltavskaya et al. Life (Basel) 2021; 11(10) 997).

**Objectives:**

The aim of the study was to identify associations of the GRIN2A gene rs11644461 polymorphism with features of the course of schizophrenia.

**Methods:**

This study was carried out in accordance with the Code of Ethics of the World Medical Association (Declaration of Helsinki 1975). 805 patients with schizophrenia (ICD-10: F20) were included. Clinical examination and diagnostic evaluation were performed using the Positive and Negative Syndrome Scale (PANSS). From the general group of patients, 2 subgroups were distinguished according to the PANSS survey: 391 patients with leading negative symptoms and 414 patients with leading positive symptoms. Also 2 subgroups were distinguished from the general group of patients: 398 patients with a continuous course of schizophrenia and 257 patients with episodic schizophrenia. Genotyping was performed by real-time PCR.

**Results:**

An association of the C rs11644461 GRIN2A allele with the continuous course of schizophrenia was revealed (p<0,047). The rs11644461 polymorphism was not associated with the leading symptoms of the disease (positive or negative). At the same time, the values of the total score on the PANSS scale differed statistically significantly in carriers of different genotypes for this polymorphism. The sum of PANSS scores (Me [Q25 – Q75]) in carriers of the TC rs11644461 genotype was statistically significantly higher (106 [92–113]) than in carriers of the CC genotype (101 [87–108]) (p=0.006).

**Conclusions:**

According to the results obtained, carriers of the TC rs11644461 GRIN2A genotype have a higher severity of schizophrenia symptoms according to the PANSS scale than carriers of the CC genotype. Also in the present study, it was shown that the C allele rs11644461 GRIN2A is associated with the continuous course of schizophrenia, which indicates the contribution of this locus to the formation of the course of the disease.

**Disclosure of Interest:**

E. Poltavskaya Grant / Research support from: Russian Science Foundation # 21-15-00212, O. Fedorenko Grant / Research support from: Russian Science Foundation # 21-15-00212, E. Kornetova Grant / Research support from: Russian Science Foundation # 21-15-00212, I. Pozhidaev Grant / Research support from: Russian Science Foundation # 21-15-00212, D. Paderina Grant / Research support from: Russian Science Foundation # 21-15-00212

